# Supplementary material for: Machine Learning Accelerates Crystallization for Structure Determination
Source: Angew Chem Int Ed Engl. 2026 May 4;65(25):e1218503. doi: 10.1002/anie.1218503 (PMC13266958; doi:10.1002/anie.1218503)

checkCIF/PLATON report

Structure factors have been supplied for datablock(s) lcz035

THIS REPORT IS FOR GUIDANCE ONLY. IF USED AS PART OF A REVIEW PROCEDURE FOR PUBLICATION, IT SHOULD NOT REPLACE THE EXPERTISE OF AN EXPERIENCED CRYSTALLOGRAPHIC REFEREE.

No syntax errors found. CIF dictionary Interpreting this report

Datablock: lcz035

Bond precision:	C-C = 0.0111 A		Wavelength=1.54184
Cell:	a=22.7183(2)	b=13.0496(1)	c=24.3237(4)
	alpha=90	beta=108.152(1)	gamma=90
Temperature:	100 K		

	Calculated	Reported
Volume	6852.25(14)	6852.24(14)
Space group	P 21/c	P 1 21/c 1
Hall group	-P 2ybc	-P 2ybc
Moiety formula	C15 H3 Ag3 F18 N6, C11 H14 O3	C15 H3 Ag3 F18 N6, C11 H14 O3
Sum formula	C26 H17 Ag3 F18 N6 O3	C26 H17 Ag3 F18 N6 O3
Mr	1127.07	1127.06
Dx,g cm-3	2.185	2.185
Z	8	8
Mu (mm-1)	14.928	14.928
F000	4336.0	4336.0
F000'	4356.96	
h,k,lmax	28,16,30	28,16,29
Nref	14742	14167
Tmin,Tmax	0.126,0.051	0.344,1.000
Tmin'	0.020	

Correction method= # Reported T Limits: Tmin=0.344 Tmax=1.000
AbsCorr = MULTI-SCAN

Data completeness= 0.961 Theta(max)= 78.532

R(reflections)= 0.0544(12037) wR2(reflections)= 0.1598(14167)

S = 1.116 Npar= 1015

The following ALERTS were generated. Each ALERT has the format
test-name_ALERT_alert-type_alert-level.
Click on the hyperlinks for more details of the test.

Alert level C

```
PLAT213_ALERT_2_C Atom F01V            has ADP max/min Ratio .....        3.4 prolat
PLAT242_ALERT_2_C Low    'MainMol' Ueq as Compared to Neighbors of       C033 Check
PLAT250_ALERT_2_C Large U3/U1 Ratio for <U(i,j)> Tensor(Resd    1)        2.1 Note
PLAT250_ALERT_2_C Large U3/U1 Ratio for <U(i,j)> Tensor(Resd    2)        2.1 Note
PLAT342_ALERT_3_C Low Bond Precision on  C-C Bonds ..............    0.01109 Ang.
PLAT906_ALERT_3_C Large K Value in the Analysis of Variance ......      2.386 Check
PLAT911_ALERT_3_C Missing FCF Refl Between Thmin & STh/L=    0.600         25 Report
                6  7  0,   9  5  2,   0 10  3,   4  7  3, -13  1  4, -18 10  5,
              -12  3  5,  -4 11  5,  24  1  5, -12  3  6,  16 10  8, -22  9 11,
              -20 10 12,   1  3 13,   2  3 13,   1  3 15,  11  7 16, -11  0 20,
              -19  6 23, -17  0 24, -13  0 28, -12  0 28, -11  0 28, -10  0 28,
               -9  0 28,
PLAT971_ALERT_2_C Check Calcd Resid. Dens.  0.84Ang From Ag01          2.39 eA-3
PLAT971_ALERT_2_C Check Calcd Resid. Dens.  1.11Ang From Ag05          2.38 eA-3
PLAT971_ALERT_2_C Check Calcd Resid. Dens.  0.89Ang From Ag02          2.37 eA-3
PLAT971_ALERT_2_C Check Calcd Resid. Dens.  1.05Ang From Ag02          2.28 eA-3
PLAT971_ALERT_2_C Check Calcd Resid. Dens.  0.84Ang From Ag04          2.25 eA-3
PLAT971_ALERT_2_C Check Calcd Resid. Dens.  0.93Ang From Ag03          2.24 eA-3
PLAT971_ALERT_2_C Check Calcd Resid. Dens.  0.95Ang From Ag03          2.21 eA-3
PLAT971_ALERT_2_C Check Calcd Resid. Dens.  0.97Ang From Ag06          2.21 eA-3
PLAT971_ALERT_2_C Check Calcd Resid. Dens.  1.17Ang From Ag04          2.18 eA-3
PLAT971_ALERT_2_C Check Calcd Resid. Dens.  0.91Ang From Ag05          2.17 eA-3
PLAT971_ALERT_2_C Check Calcd Resid. Dens.  1.00Ang From Ag01          2.16 eA-3
PLAT971_ALERT_2_C Check Calcd Resid. Dens.  1.01Ang From Ag06          2.06 eA-3
PLAT972_ALERT_2_C Check Calcd Resid. Dens.  0.78Ang From Ag01         -1.69 eA-3
PLAT972_ALERT_2_C Check Calcd Resid. Dens.  0.76Ang From Ag06         -1.69 eA-3
PLAT972_ALERT_2_C Check Calcd Resid. Dens.  0.75Ang From Ag01         -1.67 eA-3
PLAT972_ALERT_2_C Check Calcd Resid. Dens.  0.78Ang From Ag05         -1.60 eA-3
PLAT972_ALERT_2_C Check Calcd Resid. Dens.  0.71Ang From Ag05         -1.60 eA-3
PLAT972_ALERT_2_C Check Calcd Resid. Dens.  0.73Ang From Ag03         -1.56 eA-3
PLAT972_ALERT_2_C Check Calcd Resid. Dens.  0.69Ang From Ag02         -1.52 eA-3
PLAT972_ALERT_2_C Check Calcd Resid. Dens.  0.74Ang From Ag02         -1.52 eA-3
```

Alert level G

```
PLAT007_ALERT_5_G Number of Unrefined Donor-H Atoms .............          2 Report
              H00T  H01F
PLAT083_ALERT_2_G SHELXL Second Parameter in WGHT  Unusually Large      17.79 Why ?
PLAT142_ALERT_4_G s.u. on b - Axis Small or Missing .............    0.00010 Ang.
PLAT242_ALERT_2_G Low    'MainMol' Ueq as Compared to Neighbors of       C01X Check
PLAT242_ALERT_2_G Low    'MainMol' Ueq as Compared to Neighbors of       C022 Check
PLAT242_ALERT_2_G Low    'MainMol' Ueq as Compared to Neighbors of       C02J Check
```

```
PLAT242_ALERT_2_G Low    'MainMol' Ueq as Compared to Neighbors of       C02L Check
PLAT242_ALERT_2_G Low    'MainMol' Ueq as Compared to Neighbors of       C02M Check
PLAT242_ALERT_2_G Low    'MainMol' Ueq as Compared to Neighbors of       C02Q Check
PLAT242_ALERT_2_G Low    'MainMol' Ueq as Compared to Neighbors of       C026 Check
PLAT242_ALERT_2_G Low    'MainMol' Ueq as Compared to Neighbors of       C029 Check
PLAT242_ALERT_2_G Low    'MainMol' Ueq as Compared to Neighbors of       C02G Check
PLAT242_ALERT_2_G Low    'MainMol' Ueq as Compared to Neighbors of       C02I Check
PLAT242_ALERT_2_G Low    'MainMol' Ueq as Compared to Neighbors of       C02N Check
PLAT242_ALERT_2_G Low    'MainMol' Ueq as Compared to Neighbors of       C02W Check
PLAT434_ALERT_2_G Short Inter HL..HL Contact F00C      ..F00L      .        2.84 Ang.
                                             2-x,2-y,1-z  =      3_776 Check
PLAT434_ALERT_2_G Short Inter HL..HL Contact F018      ..F01W      .        2.77 Ang.
                                                   x,y,z  =      1_555 Check
PLAT720_ALERT_4_G Number of Unusual/Non-Standard Labels ..........         146 Note
              Ag01    Ag02    Ag03    Ag04    Ag05    Ag06    F007    F008
              F009    F00A    F00B    F00C    F00D    F00E    F00F    F00G
              F00H    O00I    F00J    F00K    F00L    F00M    F00N    F00O
              F00P    F00Q    F00R    F00S    O00T    H00T    F00U    F00V
              N00W    N00X    N00Y    F00Z    N010    F011    F012    F013
              F014    N015    N016    F017    F018    N019    N01A    N01B
              N01C    F01D    F01E    O01F    H01F    N01G    F01H    O01I
              N01J    C01K    F01L    O01M    C01N    C01O    C01P    C01Q
              C01R    H01R    C01S    H01S    C01T    H01T    C01U    F01V
              F01W    C01X    C01Y    H01Y    C01Z    C020    C021    C022
              C023    H023    C024    C025    C026    C027    C028    H028
              C029    C02A    C02B    C02C    H02A    H02B    C02D    H02D
              C02E    C02F    H02F    C02G    C02H    H02C    H02E    H02G
              C02I    C02J    C02K    H02K    C02L    C02M    C02N    C02O
              H02H    H02I    C02P    C02Q    C02R    H02R    C02S    H02J
              H02L    H02M    C02T    C02U    H02U    C02V    H02V    C02W
              C02X    C02Y    C02Z    H02N    H02O    C030    H03A    H03B
              C031    H03C    H03D    H03E    O032    C033    C034    H03F
              H03G    H03H
PLAT883_ALERT_1_G Absent Datum for _atom_sites_solution_primary ..      Please Do !
PLAT910_ALERT_3_G Missing FCF Reflection(s) Below Theta(Min)[Deg]=       3.73 Note
                1  0  0,
PLAT912_ALERT_4_G Missing # of FCF Reflections Above STh/L=  0.600         501 Note
PLAT941_ALERT_3_G Average HKL Measurement Multiplicity ..........          2.7 Low
PLAT969_ALERT_5_G The 'Henn et al.' R-Factor-gap value ..........        2.875 Note
              Predicted wR2: Based on SigI**2  5.56 or SHELX Weight 14.33
PLAT978_ALERT_2_G Number C-C Bonds with Positive Residual Density.           0 Info
```

---

0 **ALERT level A** = Most likely a serious problem - resolve or explain
0 **ALERT level B** = A potentially serious problem, consider carefully
27 **ALERT level C** = Check. Ensure it is not caused by an omission or oversight
24 **ALERT level G** = General information/check it is not something unexpected

1 ALERT type 1 CIF construction/syntax error, inconsistent or missing data
40 ALERT type 2 Indicator that the structure model may be wrong or deficient
5 ALERT type 3 Indicator that the structure quality may be low
3 ALERT type 4 Improvement, methodology, query or suggestion
2 ALERT type 5 Informative message, check

Validation response form

Please find below a validation response form (VRF) that can be filled in and pasted into your CIF.

```
# start Validation Reply Form
_vrf_PLAT213_lcz035
;
PROBLEM: Atom F01V            has ADP max/min Ratio .....        3.4 prolat
RESPONSE: ...
;
_vrf_PLAT242_lcz035
;
PROBLEM: Low    'MainMol' Ueq as Compared to Neighbors of       C033 Check
RESPONSE: ...
;
_vrf_PLAT250_lcz035
;
PROBLEM: Large U3/U1 Ratio for <U(i,j)> Tensor(Resd    1)        2.1 Note
RESPONSE: ...
;
_vrf_PLAT342_lcz035
;
PROBLEM: Low Bond Precision on  C-C Bonds ..............    0.01109 Ang.
RESPONSE: ...
;
_vrf_PLAT906_lcz035
;
PROBLEM: Large K Value in the Analysis of Variance ......      2.386 Check
RESPONSE: ...
;
_vrf_PLAT911_lcz035
;
PROBLEM: Missing FCF Refl Between Thmin & STh/L=    0.600         25 Report
RESPONSE: ...
;
_vrf_PLAT971_lcz035
;
PROBLEM: Check Calcd Resid. Dens.  0.84Ang From Ag01           2.39 eA-3
RESPONSE: ...
;
_vrf_PLAT972_lcz035
;
PROBLEM: Check Calcd Resid. Dens.  0.78Ang From Ag01          -1.69 eA-3
RESPONSE: ...
;
# end Validation Reply Form
```

It is advisable to attempt to resolve as many as possible of the alerts in all categories. Often the minor alerts point to easily fixed oversights, errors and omissions in your CIF or refinement strategy, so attention to these fine details can be worthwhile. It is up to the individual to critically assess their own results and, if necessary, seek expert advice.

PLATON version of 17/09/2025; check.def file version of 17/09/2025

---

duplicate check

No duplication found

Datablock lcz035 - ellipsoid plot

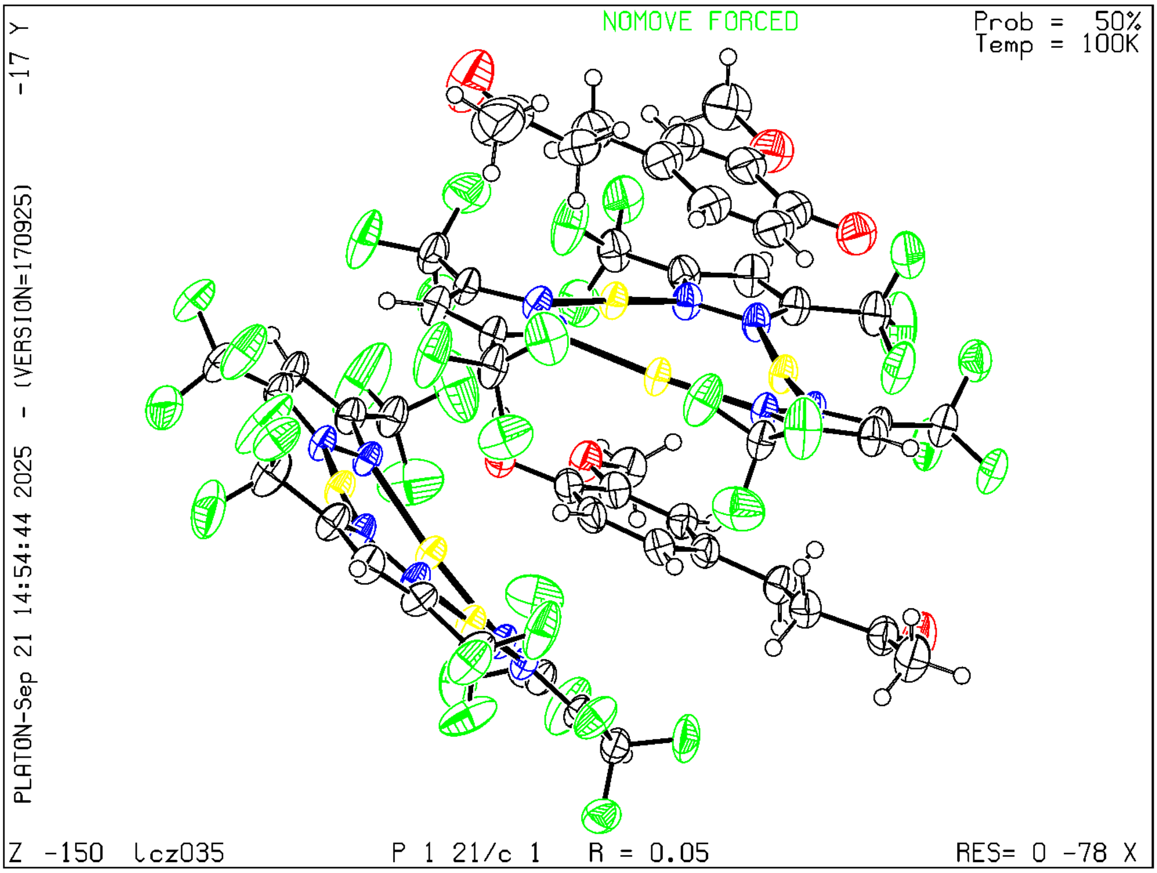

Supplement: Supplementary file 2 — Supporting File 2: anie72462‐sup‐0002‐DataFile.zip. [file ANIE-65-e1218503-s002.zip › supplementary_materials_crystallographic_data submit.zip!/cif_and_checkcif(1-114)/Ag3Pz3_35/Ag3Pz3_35_checkcif.pdf]
